# Comparison Between Two Definitions of Contrast-Associated Acute Kidney Injury in Patients With Congestive Heart Failure

**DOI:** 10.3389/fcvm.2022.763656

**Published:** 2022-04-27

**Authors:** Bo Wang, Yiying Zheng, Huanqiang Li, Shuling Chen, Ziyou Zhou, Zhubin Lun, Ming Ying, Lingyu Zhang, Ziling Mai, Liwei Liu, Ziqing Zhou, Mengfei Lin, Yongquan Yang, Jiyan Chen, Yong Liu, Jin Liu, Shiqun Chen, Ning Tan

**Affiliations:** ^1^Department of Cardiology, Guangdong Provincial People's Hospital, Guangdong Cardiovascular Institute, Guangdong Academy of Medical Sciences, Guangzhou, China; ^2^Guangdong Provincial Key Laboratory of Coronary Heart Disease Prevention, Guangdong Provincial People's Hospital, Guangdong Cardiovascular Institute, Guangdong Academy of Medical Sciences, Guangzhou, China; ^3^The Second School of Clinical Medicine, Southern Medical University, Guangzhou, China; ^4^Department of Cardiology, People's Hospital of Yangjiang, Yangjiang, China; ^5^School of Medicine, Guangdong Provincial People's Hospital, South China University of Technology, Guangzhou, China; ^6^The First School of Clinical Medicine, Guangdong Medical University, Zhanjiang, China; ^7^Department of Cardiology, Maoming People's Hospital, Maoming, China; ^8^School of Pharmacy, Guangdong Pharmaceutical University, Guangzhou, China

**Keywords:** contrast-associated acute kidney injury (CA-AKI), congestive heart failure (CHF), long-term all-cause mortality, population attributable risk (PAR), coronary angiography (CAG)

## Abstract

**Background:**

Different definitions of contrast-associated acute kidney injury (CA-AKI) have different predictive effects on prognosis. However, few studies explored the relationship between these definitions and long-term prognosis in patients with congestive heart failure (CHF). Thus, we aimed to evaluate this association and compared the population attributable risks (PAR) of different CA-AKI definitions.

**Methods:**

This study enrolled 2,207 consecutive patients with CHF undergoing coronary angiography (CAG) in Guangdong Provincial People's Hospital. Two different definitions of CA-AKI were used: CA-AKI_A_ was defined as an increase ≥.5 mg/dl or > 25% in serum creatinine (SCr) from baseline within 72 h after CAG, and CA-AKI_B_ was defined as an increase of ≥.3 mg/dl or > 50% in SCr from baseline within 48 h after CAG. Kaplan-Meier methods and Cox regression were applied to evaluate the association between CA-AKI with long-term mortality. Population attributable risk (PAR) of different definitions for long-term prognosis was also calculated.

**Results:**

During the 3.8-year median follow-up (interquartile range 2.1-6), the overall long-term mortality was 24.9%, and the long-term mortality in patients with the definitions of CA-AKI_A_ and CA-AKI_B_ were 30.4% and 34.3%, respectively. We found that CA-AKI_A_ (HR: 1.44, 95% CI 1.19-1.74) and CA-AKI_B_ (HR: 1.48, 95% CI 1.21-1.80) were associated with long-term mortality. The PAR was higher for CA-AKI_A_ (9.6% vs. 8%).

**Conclusions:**

Our findings suggested that CA-AKI was associated with long-term mortality in patients with CHF irrespective of its definitions. The CA-AKI_A_ was a much better definition of CA-AKI in patients with CHF due to its higher PAR. For these patients, cardiologists should pay more attention to the presence of CA-AKI, especially CA-AKI_A_.

## Introduction

As the common postoperative complication, contrast-associated acute kidney injury (CA-AKI) can significantly increase the risk of adverse clinical outcomes, including prolonged hospitalization, adverse cardiovascular events, renal diseases, and even long-term mortality (1–3). Previous studies proved that congestive heart failure (CHF) was an independent risk factor of CA-AKI, and patients with CHF were 1–2 times more likely to develop CA-AKI than those without CHF (4, 5).

In general, changes in plasma creatinine levels were used to diagnose the CA-AKI. The criterion is usually incremented in serum creatinine (SCr) concentration of at least 0.5 mg/dl or more than 25% within 72 h of contrast media exposure (6). However, according to some other studies, CA-AKI was confirmed as of this criterion: SCr levels increase more than 0.3 mg/dl or 50% after exposure to contrast media within 48 hours (7). A previous study found that different prevalence appeared on different definitions of CA-AKI (8). The previous study found different definition has different effect to predict long-term prognosis among patients with AMI and diabetes mellitus according to population attributable risk (PAR) (9, 10).

However, to our acknowledge, few papers evaluated the association between long-term all-cause mortality and various CA-AKI definitions among patients with CHF. Therefore, this study intended to assess the association between different CA-AKI definitions and long-term prognosis among patients with CHF receiving coronary angiography (CAG) and to determine the appropriate definition of CA-AKI in these patients.

## Method

### Study Design and Population

**Figure 1:**
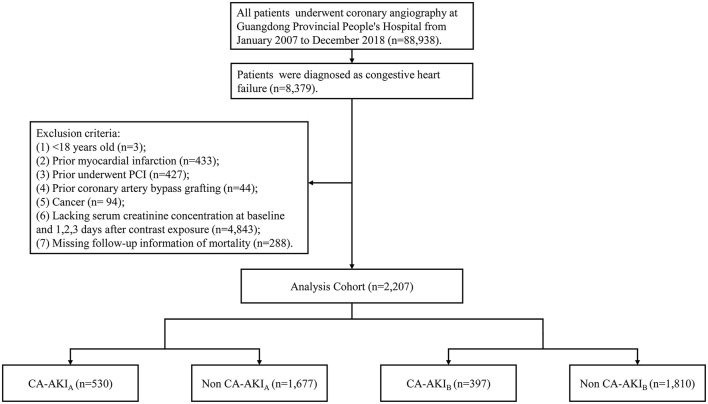
Study flow chart.

This study used the Cardiorenal Improvement study data, which was carried out at the largest cardiovascular center in Southern China (Guangdong Provincial People's Hospital, China, Clinicaltrials.gov NCT04407936). The Cardiorenal Improvement study extracted demographic characteristics, comorbidities, laboratory tests, and discharge medication from the institution's electronic medical record system. All patients received the percutaneous coronary intervention (PCI) and CAG by standard guidelines (11–14). The data quality control, as well as regular data verification, were placed in charge of senior cardiologists. The follow-up information of all patients was retrieved from the Public Security System of Guangdong province. All patients receiving CAG were screened between Jan. 1, 2007 and Dec. 31, 2018. A total of 88,938 patients received CAG, while 8,379 patients were confirmed as CHF. The flow chart including inclusion criteria and exclusion criteria of the current study was shown in Figure 1. The study protocol was developed in accordance with the Declaration of Helsinki and was authorized by the Ethics Committee of Guangdong Provincial People's Hospital (No. GDREC2019555H[R1]).

### Endpoint and Definitions

The primary study outcome was long-term all-cause mortality. CHF was defined as Killip classes II-IV or New York Heart Association (NYHA) classes III-IV (4). The definitions of CA-AKI_A_ and CA-AKI_B_ are SCr concentration increasing more than 0.5 mg/dl or 25% within 72 h postoperatively (6), and SCr level increasing more than 0.3 mg/dl or 50% within 48 h postoperatively, respectively (7). Comorbidities included coronary artery disease (CAD), acute myocardial infarction (AMI), hypertension, chronic kidney disease (CKD), anemia, diabetes mellitus, atrial fibrillation, chronic obstructive pulmonary disease (COPD), and stroke. This study used results of CAG and the 10th revision (ICD-10) of the International Classification of Diseases to determine CAD. Anemia was confirmed by gender and hematocrit (male's hematocrit below 39% and female's hematocrit below 36%) (15). Our study calculated the estimated glomerular filtration rate (eGFR) by applying the Modification of Diet in Renal Disease (MDRD) formula (16). The definition of CKD was eGFR ≤60 mL/min/1.73 m^2^ (17, 18).

### Statistical Analysis

Normally distributed data, abnormally distributed data, and categorical data are presented as mean (standard deviation [SD]), (interquartile range [IQR]), as well as number (percentage). The difference between patients with and without CA-AKI was tested by Student's *t*-test, Wilcoxon rank-sum tests, and Chi-square test for continuous variables with normal distribution, continuous variables with abnormal distribution, and categorical variables. Kaplan-Meier method and log-rank test were applied to identify the prognostic differences. The association between CA-AKI and outcomes was evaluated by using the Cox proportional-hazards model, and presented as hazard ratio (HR) and 95% confidence interval (CI). Covariates included age, sex, CAD, diabetes mellitus, CKD, hypertension, hypoalbuminemia, anemia, COPD, and stroke. Two multivariate Cox regression models have been constructed for CA-AKI_A_ and CA-AKI_B_ accordingly. The PAR was calculated on the basis of the following formula: PAR = prevalence ^*^ (hazard ratio - 1)/ [1 + prevalence ^*^ (hazard ratio – 1)]. The prevalence is the prevalence of CA-AKI_A_ and CA-AKI_B_ in our study. The present study applied the delta method to estimate the PAR's standard error. The present study used R software (R Foundation for Statistical Computing), version 3.6.3 to carry out statistical analyses. All p-values less than.05 were considered statistically significant.

## Result

The present study eventually enrolled 2,207 patients. Table 1 showed the baseline characteristics. In general, the mean age was 64.3 ± 10.7 years, and 68.7% of the patients were male. The number of patients complicated with CAD, hypertension, diabetes mellitus, and CKD was 1,529 (69.3%), 1,090 (49.4%), 6,92 (31.4%), and 995 (45.1%), respectively. A total of 1,153 (52.2%) patients underwent PCI. The mean of eGFR was 63.3 ± 27.1 mL/min/1.73 m^2^.

**Table 1:** Baseline characteristics.

Characteristic^*^		**CA-AKI**_**A**_			**CA-AKI**_**B**_		
	**Overall**	**Non-CA-AKI_**A**_**	**CA-AKI_**A**_**	***P value***	**Non-CA-AKI_**B**_**	**CA-AKI_**B**_**	***P value***
	**(*n =* 2,207)**	**(*n =* 1,677)**	**(*n =* 530)**		**(*n =* 1,810)**	**(*n =* 397)**	
**Demographic characteristics**						
age, year	64.3 (10.7)	64.2 (10.8)	64.6 (10.3)	0.44	64.1 (10.7)	65.4 (10.5)	0.03
Age ≥ 75 years, n (%)	416 (18.9)	321 (19.1)	95 (17.9)	0.58	332 (18.3)	84 (21.2)	0.22
Male, n (%)	1,517 (68.7)	1,210 (72.2)	307 (57.9)	<0.001	1259 (69.6)	258 (65.0)	0.09
**Coexisting conditions**							
CAD, n (%)	1,529 (69.3)	1,234 (73.6)	295 (55.7)	<0.001	1,277 (70.6)	252 (63.5)	0.007
PCI, n (%)	1,153 (52.2)	952 (56.8)	201 (37.9)	<0.001	984 (54.4)	169 (42.6)	<0.001
AMI, n (%)	796 (36.1)	663 (39.5)	133 (25.1)	<0.001	689 (38.1)	107 (27.0)	<0.001
Hypertension, n (%)	1,090 (49.4)	854 (50.9)	236 (44.5)	0.01	890 (49.2)	200 (50.4)	0.70
Diabetes mellitus, n (%)	692 (31.4)	537 (32.0)	155 (29.3)	0.25	559 (30.9)	133 (33.5)	0.34
CKD, n (%)	995 (45.1)	773 (46.1)	222 (41.9)	0.1	779 (43.0)	216 (54.4)	<0.001
Anemia, n (%)	1,056 (48.0)	758 (45.4)	298 (56.2)	<0.001	812 (45.0)	244 (61.5)	<0.001
Hypoalbuminemia, n (%)	1245 (57.7)	951 (58.1)	294 (56.2)	0.47	1008 (57.0)	237 (60.6)	0.21
Atrial fibrillation, n (%)	387 (17.5)	252 (15.0)	135 (25.5)	<0.001	300 (16.6)	87 (21.9)	0.01
COPD, n (%)	33 (1.5)	27 (1.6)	6 (1.1)	0.56	29 (1.6)	4 (1.0)	0.51
Stroke, n (%)	183 (8.3)	130 (7.8)	53 (10.0)	0.12	136 (7.5)	47 (11.8)	0.006
**Laboratory examination**							
Total cholesterol, mmol/L	4.5 (1.2)	4.5 (1.2)	4.6 (1.1)	0.08	4.5 (1.2)	4.6 (1.2)	0.15
HDL-C, mmol/L	1.0 (0.3)	1.0 (0.3)	1.0 (0.3)	<0.001	1.0(0.3)	1.0 (0.3)	0.27
LDL-C, mmol/L	2.9 (1.0)	2.9 (1.0)	2.9 (0.9)	0.56	2.9 (1.0)	2.9 (1.0)	0.37
Albumin, g/L	33.8 (4.9)	33.8 (4.7)	33.9 (5.4)	0.77	34.0 (4.8)	33.3 (5.5)	0.01
eGFR, mL/min/1.73 m^2^	63.3 (27.1)	62.3 (25.8)	66.6 (30.5)	0.001	64.4 (26.5)	58.2 (29.1)	<0.001
**Medicine**							
RASi, n (%)	797 (39.2)	680 (42.7)	117 (26.4)	<0.001	712 (41.5)	85 (26.5)	<0.001
β-blocker, n (%)	1,420 (69.7)	1,163 (73.0)	257 (58.0)	<0.001	1,224 (71.4)	196 (61.1)	<0.001
Statins, n (%)	1,373 (67.4)	1,157 (72.6)	216 (48.7)	<0.001	1,189 (69.3)	184 (57.3)	<0.001
**Event**							
Long-term all-cause death, n (%)	549 (24.9)	388 (23.1)	161 (30.4)	0.001	413 (22.8)	136 (34.3)	<0.001

* *Data are presented as the mean value (standard deviation), median [interquartile range], or a number of participants (percentage)*.

*CAD, coronary artery disease; PCI, percutaneous coronary intervention; AMI, acute myocardial infarction; CKD, chronic kidney disease; COPD, chronic obstructive pulmonary disease; HDL-C, high-density lipoprotein cholesterol; LDL-C, low-density lipoprotein cholesterol; RASi, renin-angiotensin system inhibitor*.

According to the CA-AKI_A_ criteria, CA-AKI occurred in 530 patients (24%), while according to the CA-AKI_B_, CA-AKI occurred in 397 patients (18%). Results of demographic characteristics showed that patients with CA-AKI_B_ were younger compared with patients without CA-AKI_B_. However, there was no significant difference in age between patients with and without CA-AKI_A_. Additionally, patients with CA-AKI_A_ were less often male. About comorbidities, irrespective of the definition used, patients with CA-AKI complications following CAG had lower proportions of CAD, AMI, and PCI. However, patients with CA-AKI had a higher incidence of anemia, atrial fibrillation, and better renal function. Furthermore, the usage of renin-angiotensin system inhibitor (RASi), β-blocker, and statins in patients with CA-AKI was lower, regardless of the definition of CA-AKI.

**Figure 2:**
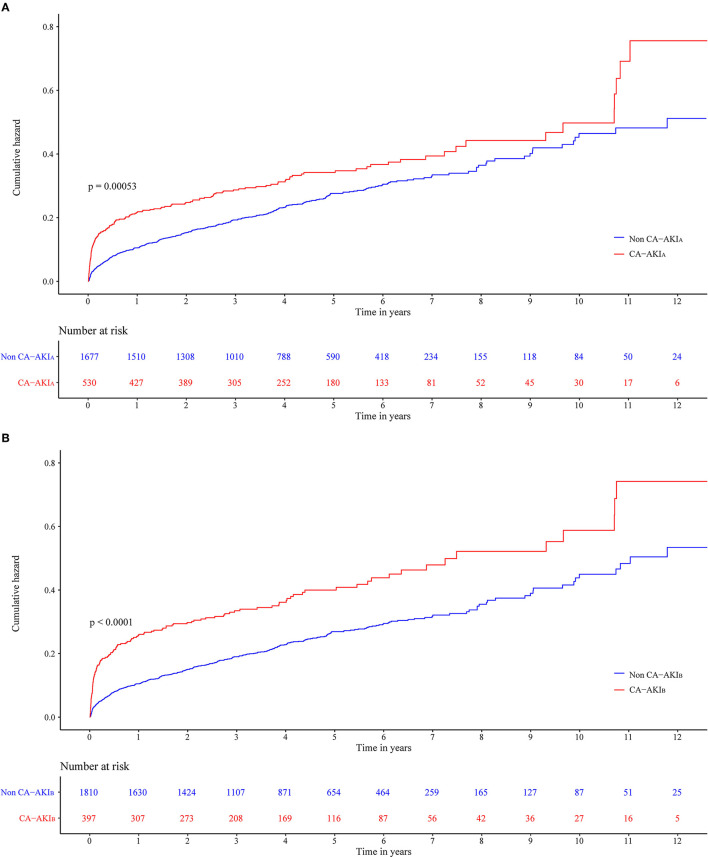
Cumulative incidence of all-cause death for two different contrast-associated acute kidney injury (CA-AKI) definitions patients with congestive heart failure (CHF). **(A)** Definition of CA-AKI_A_. **(B)** Definition of CA-AKI_B_.

**Figure 3:**
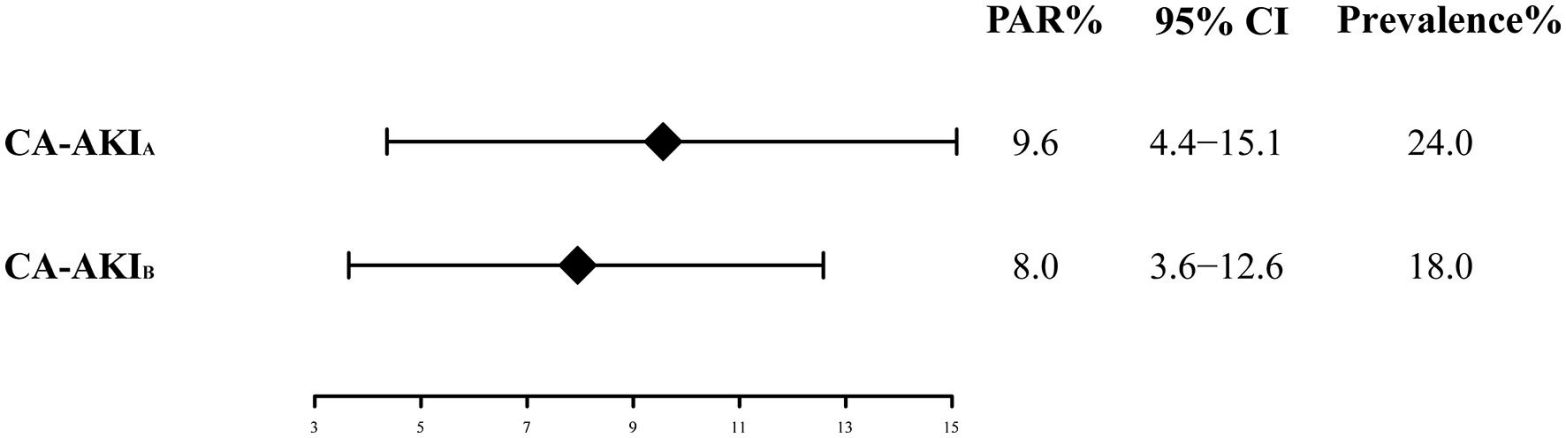
Population attributable risk of two different definitions of CA-AKI.

The long-term all-cause mortality was 24.9% during the follow-up period, whose median was 3.8 (IQR 2.1-6) years. Among patients with CA-AKI_A_ or CA-AKI_B_, the mortalities were 30.4% and 34.3%, respectively. Kaplan-Meier curves illustrate that those patients developing CA-AKI had a worse prognosis, irrespective of the CI-AKI definition (Figure 2). Table 2 displayed the results of the univariate and multivariate cox regression. After adjusting for confounders, CA-AKI was associated with a worse prognosis. Both CA-AKI_A_ and CA-AKI_B_ could increase the risk of long-term death by 44 and 48%, (adjusted HR 1.44, 95% CI 1.19–1.74; adjusted HR 1.48, 95% CI 1.21–1.80). By evaluating the performance of the two multivariable models including CA-AKI_A_ and CA-AKI_B_, the C-statistic showed no significant difference between the two models (0.659 vs. 0.660; *p* = 0.55; Table 3). The prevalence of CA-AKI_A_ was higher (24% vs. 18%). For the PAR, it was also higher for CA-AKI_A_ (PAR: 9.6%, CI: 4.4-15.1%). The PAR for CA-AKI_B_ was 8% (CI: 3.6-12.6%) (Figure 3).

**Table 2:** Unadjusted and adjusted hazard ratios (HRs) and 95% CIs for the primary endpoint (long-term all-cause mortality) of two different contrast-associated acute kidney injury definitions.

	**Univariate Cox regression**			**Multivariate Cox regression**					
				**CA-AKI**_**A**_			**CA-AKI**_**B**_		
	**HR**	**95% CI**	***p*-value**	**HR**	**95% CI**	***p*-value**	**HR**	**95% CI**	***p*-value**
CA–AKI_A_	1.38	1.15–1.66	0.001	1.44	1.19-1.74	<0.001			
CA–AKI_B_	1.66	1.37–2.01	<0.001				1.48	1.21-1.80	<0.001
Age	1.03	1.02–1.04	<0.001	1.01	1.01-1.02	0.002	1.01	1.01-1.02	0.001
Male	1.10	0.91–1.32	0.33	1.11	0.91-1.34	0.31	1.08	0.89-1.31	0.43
CAD	1.54	1.26–1.87	<0.001	1.08	0.86-1.36	0.51	1.06	0.84-1.33	0.65
PCI	0.99	0.84–1.17	0.88						
AMI	0.98	0.83–1.17	0.85						
Hypertension	1.45	1.22–1.71	<0.001	1.05	0.87-1.27	0.60	1.05	0.87-1.27	0.62
Diabetes mellitus	1.48	1.25–1.76	<0.001	1.19	0.98-1.43	0.08	1.19	0.98-1.44	0.07
CKD	2.06	1.74–2.45	<0.001	1.64	1.36-1.98	<0.001	1.58	1.31-1.91	<0.001
Anemia	1.77	1.49–2.10	<0.001	1.27	1.05-1.53	0.01	1.26	1.05-1.53	0.02
Hypoalbuminemia	1.71	1.42–2.06	<0.001	1.35	1.11-1.65	0.003	1.36	1.11-1.65	0.002
COPD	1.74	1.04–2.91	0.03	1.85	1.10-3.11	0.02	1.86	1.11-3.13	0.02
Stroke	1.96	1.53–2.51	<0.001	1.65	1.27-2.14	<0.001	1.64	1.26-2.12	<0.001
Atrial fibrillation	0.95	0.75–1.19	0.63						

*CA-AKI_A_, defined as an increase ≥.5 mg/dl or > 25% in serum creatinine (SCr) from baseline within 72 h after CAG; CA-AKI_B_, defined as an increase ≥.3 mg/dl or > 50% in SCr from baseline within 48 h after CAG; CAD, coronary artery disease; PCI, percutaneous coronary intervention; AMI, acute myocardial infarction; CKD, chronic kidney disease; COPD, chronic obstructive pulmonary disease*.

**Table 3:** C-statistics for two multivariate Cox proportional hazards models.

	**Definition of CA-AKI**_**A**_		**Definition of CA-AKI**_**B**_		
	**C-statistics**	**(95% CI)**	**C-statistics**	**(95% CI)**	***p*** **-value**
**Long-term all-cause mortality**	0.659	0.635–0.684	0.660	0.636–0.684	0.55

*CA-AKIA, defined as an increase ≥ 0.5 mg/dl or > 25% in serum creatinine (SCr) from baseline within 72 h after CAG; CA-AKIB, defined as an increase ≥ 0.3 mg/dl or > 50% in SCr from baseline within 48 h after CAG*.

## Discussion

Our study is the first to assess the relation between CA-AKI and long-term prognosis among patients with CHF undergoing CAG and compare the PAR of two different CA-AKI definitions. According to our findings, we found that patients developing CA-AKI had a lower survival rate. The mortality of CA-AKI_A_ was lower than that of CA-AKI_B_, but the prevalence was higher. After adjusting for demography and baseline comorbidities, both CA-AKI_A_ and CA-AKI_B_ could increase the risk of all-cause mortality. Comprehensively considering the HR and incidence, CA-AKI_A_ had a higher PAR, suggesting that the definition of CA-AKI_A_ was more suitable for patients with CHF.

Previous studies demonstrated that both CA-AKI_A_ and CA-AKI_B_ were associated with poor long-term outcomes. Sun et al. explored the impact of CA-AKI_A_ on all-cause long-term mortality in 696 patients with AMI undergoing PCI (19). They demonstrated that CA-AKI_A_ independently predicted long-term mortality following PCI. The CA-AKI_A_ increases the risk of all-cause death by 1.97 times. According to Centola et al.'s study, enrolling 406 patients who were complicated with ST-segment elevation myocardial infarction (STEMI) and underwent primary PCI treatment, results detected a significant association between two CA-AKI definitions and poor clinical outcomes (8).

The CHF was an established risk factor of CA-AKI (4, 5). The CHF patients were at high risk of CA-AKI. Compared with the general population, patients with CHF had a significantly higher incidence of CA-AKI. The result of an observational study conducted by Tsai et al., enrolling 985,737 patients, showed that among patients undergoing PCI treatment, the prevalence of CA-AKI was 7.1% (20). Meanwhile, Chen et al.'s study, which included patients with CHF found that 16.7% of patients experienced CA-AKI after CAG (21), which was more than twice that of the general population. Due to inconsistencies in the definition of CAAKI, its incidence ranges from 12.07 to 17.45% in patients with heart failure, while undergoing coronary angiography according to previous studies. Chen et al.'s study, which enrolled 551 patients with CHF, reported that the incidence of CA-AKI_B_ was 16.70% (21). According to Lei et al.'s finding, the incidence of CA-AKI_A_ was 17.45% in 596 patients with heart failure (22). Results in Bei et al.'s study illustrated that the rate of CA-AKI_A_ was 12.79% among patients who were diagnosed with heart failure with preserved ejection fraction (HFpEF) (23). Wang et al.'s study showed the rate of CA-AKI_B_ was 12.07% in patients who were complicated with heart failure with mid-range ejection fraction (HFmrEF) (24). Previous studies have compared the two different definitions in patients with AMI and diabetes mellitus. They all found that the PAR of CA-AKI_B_ was higher. In contrast, the result of our study found that the CA-AKI_A_'s PAR was higher.

To evaluate the relation between CA-AKI and long-term all-cause mortality, this study firstly applied the Kaplan-Meier method to observe the differences in prognosis. Then, Cox proportional hazard models were applied to adjust confounders. Next, the incidence of different CA-AKI definitions was also considered. Combining HR and morbidity, PAR was finally calculated to assess the contribution of two different definitions to long-term all-cause death. The present study demonstrated that the PAR of CA-AKI_A_ was higher, which indicate that among patients with CHF, the definition of CA-AKI_A_ was at higher risk, and the method of PAR, which is comprehensively considered a relative risk and prevalence guide to clinical practice, is worth popularizing for the direction of clinical practice.

The CA-AKI has multiple definitions that confuse clinicians about the diagnosis (25). The present study found that CA-AKI is an independent risk factor of long-term all-cause mortality among CHF patients and that the definition of CA-AKI_A_ is more suitable for patients with CHF to assess the relation between CA-AKI and prognosis than CA-AKI_B_. This helps clinicians confirm and manage high-risk patients with CHF, who are also suffering from CA-AKI. In clinical practice, more attention should be paid to patients developing CA-AKI_A_.

## Limitation

There were several limitations in this study. In the first place, the enrolled patients in the present study were patients with CHF, which may not be extrapolated to the general population, whereas our study is the first one evaluating the relation between different CA-AKI definitions and long-term all-cause deaths in these patients. Second, the data in our analysis was just from a single-center, retrospective, and observational study. Nonetheless, this center was the largest cardiac intervention center in South China. Third, it's hard to identify the progression of renal insufficiency, while some SCr data were missing during the follow-up, which may affect the results of this study. However, the present study included variables that were significantly associated with prognosis as covariates in the multivariate Cox regression analysis.

## Conclusion

Our findings proved that CA-AKI had an association with long-term all-cause mortality among patients who suffered CHF, regardless of the definition. The CA-AKI_A_ definition was determined as appropriate for patients with CHF. This can help cardiologists identify CA-AKI with a high risk of poor long-term prognosis among patients with CHF undergoing CAG.

## Data Availability Statement

The original contributions presented in the study are included in the article/Supplementary Materials, further inquiries can be directed to the corresponding author/s.

## Ethics Statement

The studies involving human participants were reviewed and approved by Research Ethics Committee of Guangdong Provincial People's Hospital, Guangdong Academy of Medical Sciences (No. GDREC2019555H[R1]). Written informed consent for participation was not required for this study in accordance with the national legislation and the institutional requirements.

## Author Contributions

YL, JL, and SC had full access to data and takes responsibility for the integrity and the accuracy of the data analysis. BW, YZ, and HL: concept and design. YY: data management. BW, YZ, HL, SC, ZL, ZiyZ, MY, LZ, ZM, LL, ZiqZ, and ML: drafting of the manuscript. YL, NT, and JC: critical revision. YL and NT: final approval to publish. All authors contributed to acquisition, analysis, interpretation of data, and read and approved the final manuscript.

## Funding

This study was supported by Beijing Lisheng Cardiovascular Health Foundation (LHJJ20141751), National Natural Science Foundation of China (Grant Nos. 81670339 and 81970311), Guangdong Provincial Science and Technology Plan Project (2017B030314041), Guangdong Medical Science and Technology Research Fund Project (No. A2022458), and Guangdong Provincial People's Hospital Dengfeng Project Fund (DFJH201919, DFJH2020011, and DFJH2020026).

## Conflict of Interest

The authors declare that the research was conducted in the absence of any commercial or financial relationships that could be construed as a potential conflict of interest.

## Publisher's Note

All claims expressed in this article are solely those of the authors and do not necessarily represent those of their affiliated organizations, or those of the publisher, the editors and the reviewers. Any product that may be evaluated in this article, or claim that may be made by its manufacturer, is not guaranteed or endorsed by the publisher.

## Abbreviations

CHF: congestive heart failure
CAG: coronary angiography
CAD: coronary artery disease
PAR: population attributable risk
PCI: percutaneous coronary intervention
AMI: previous acute myocardial infarction
CKD: chronic kidney disease
COPD: chronic obstructive pulmonary disease
HR: hazard ratio
CI: confidence interval.
